# A Case of Radical Resection of a Mediastinal Invasive Parathyroid Tumor via a Thoracoscopic Approach

**DOI:** 10.70352/scrj.cr.26-0266

**Published:** 2026-10-24

**Authors:** Yusuke Suzuki, Hiroshi Sato, Yutaka Miyawaki, Ayumi Kodama, Masatoshi Yoshizawa, Tasuku Furube, Kazuaki Matsui, Tetsuro Toriumi, Yutaka Kishimoto, Gen Ebara, Shinichi Sakuramoto

**Affiliations:** 1 Department of Digestive Surgery, Saitama Medical University International Medical Center, Hidaka, Saitama, Japan; 2 Department of Pathology, Saitama Medical University International Medical Center, Hidaka, Saitama, Japan

**Keywords:** parathyroid tumor, progression within the mediastinum, thoracoscopic surgery

## Abstract

**INTRODUCTION:**

Parathyroid carcinoma is a rare malignancy that accounts for 0.3%–5.6% of cases of primary hyperparathyroidism (PHPT). Owing to the risk of tumor seeding, biopsy and fine-needle aspiration are contraindicated, posing a challenge to preoperative diagnosis of parathyroid carcinoma. Furthermore, complete tumor resection without capsular rupture is crucial because R1 resection substantially increases the risk of local recurrence.

**CASE PRESENTATION:**

A 71-year-old woman with hypercalcemia and renal dysfunction was referred to our hospital for further evaluation. Laboratory tests revealed a serum calcium level >12 mg/dL and markedly elevated intact parathyroid hormone (iPTH) levels. CT revealed diffuse skeletal sclerosis, leading to a diagnosis of PHPT caused by a parathyroid tumor. Imaging studies demonstrated that the tumor extended from the lower right neck into the superior mediastinum, with indistinct borders from the trachea and esophagus, raising concerns regarding malignancy and possible invasion. Therefore, a thoracoscopic approach was planned to achieve safe dissection and assess the feasibility of en bloc resection. The patient was initially placed in the left lateral decubitus position, and thoracoscopic mobilization of the mediastinal portion of the tumor was performed without capsular injury. After sufficient mobilization, the patient was moved to a supine position, and cervical dissection was completed through a small incision in the neck, achieving en bloc removal while preserving the recurrent laryngeal nerve. The patient developed pneumonia postoperatively, which resolved with conservative treatment. Pathological examination of the resected tumor revealed a parathyroid adenoma with capsular invasion and negative margins. The patient was discharged on POD 9 with normalized calcium and iPTH levels.

**CONCLUSIONS:**

The thoracoscopic approach may be a useful and safe strategy for treating parathyroid tumors that extend into the mediastinum, facilitating precise mobilization and en bloc resection without capsular rupture. Hence, a combined approach may enhance both the safety and curative potential of surgery in selected cases of parathyroid tumors with mediastinal extension.

## INTRODUCTION

Primary hyperparathyroidism (PHPT) is commonly caused by parathyroid tumors. Although most of these tumors are adenomas, parathyroid carcinoma accounts for approximately 0.3%–5.6% of PHPT cases.^1–6)^ Obtaining a preoperative histological diagnosis of parathyroid carcinoma is challenging because fine-needle aspiration and other biopsy procedures are contraindicated owing to the risk of tumor seeding caused by capsular disruption.^7)^ Consequently, a definitive diagnosis is often not possible before surgery. Furthermore, because surgical resection is the standard treatment for PHPT, operative planning should always consider the possibility of malignancy.

Herein, we report a case of PHPT caused by a parathyroid tumor extending into the superior mediastinum that was successfully resected using a thoracoscopic-first approach.

## CASE PRESENTATION

A 71-year-old woman presented to a primary care physician with a 3-month history of insomnia and loss of appetite. She had no notable medical history, including no prior neck surgery. Physical examination revealed no abnormalities on inspection and palpation of the neck, and no hoarseness was observed. Laboratory testing revealed hypercalcemia and renal dysfunction, prompting referral to our hospital for further evaluation. At admission, blood laboratory testing revealed that the serum calcium (Ca), inorganic phosphorus (IP), and intact parathyroid hormone (iPTH) levels were 13.7 mg/dL, 3.3 mg/dL, and 2822 pg/mL, respectively. CT of the neck and chest revealed a large, calcified mass extending from the right thyroid lobe into the mediastinum, causing leftward displacement of the trachea and esophagus (**Fig. 1**). Methoxyisobutylisonitrile scintigraphy demonstrated radiotracer uptake corresponding to the mass. Based on these findings, the patient was diagnosed with PHPT caused by a parathyroid tumor (**Fig. 2**).

**Fig. 1:**
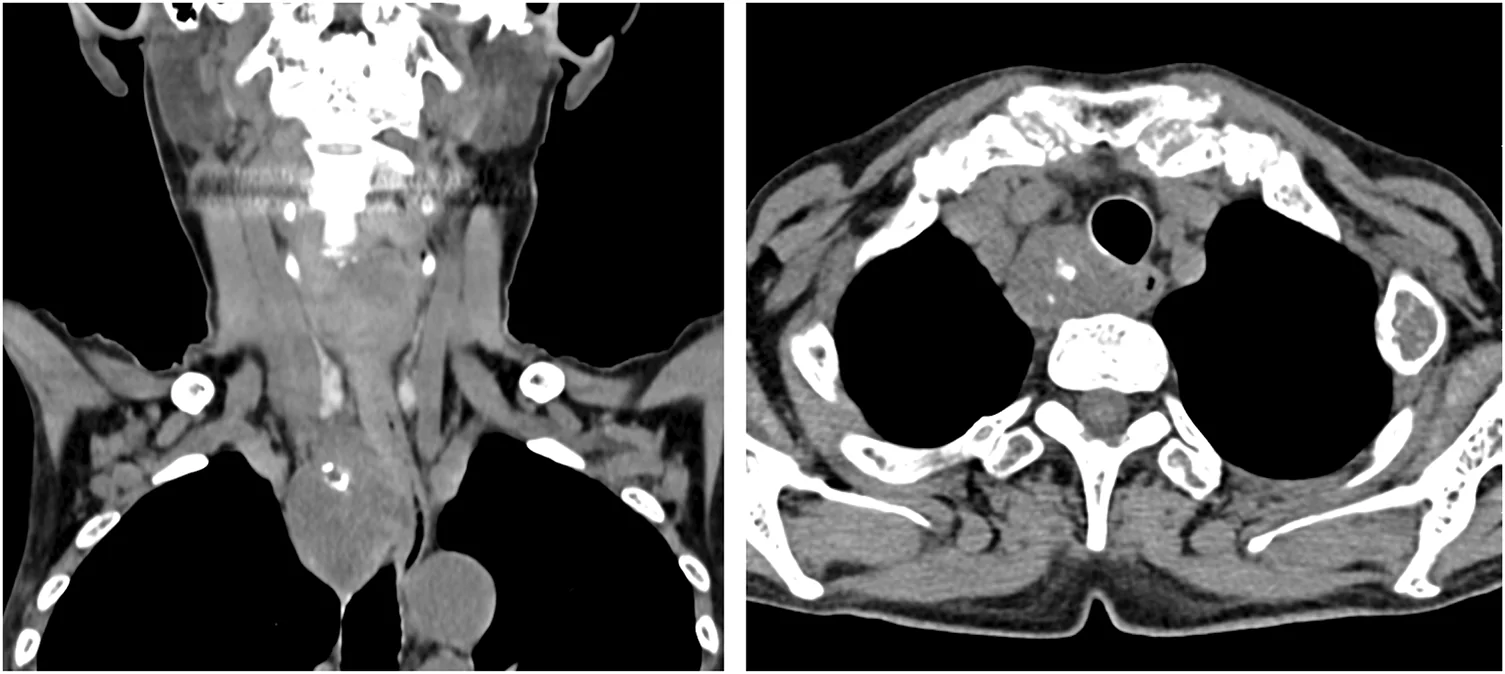
CT revealed a large calcified mass extending from the right thyroid lobe to the mediastinum.

**Fig. 2:**
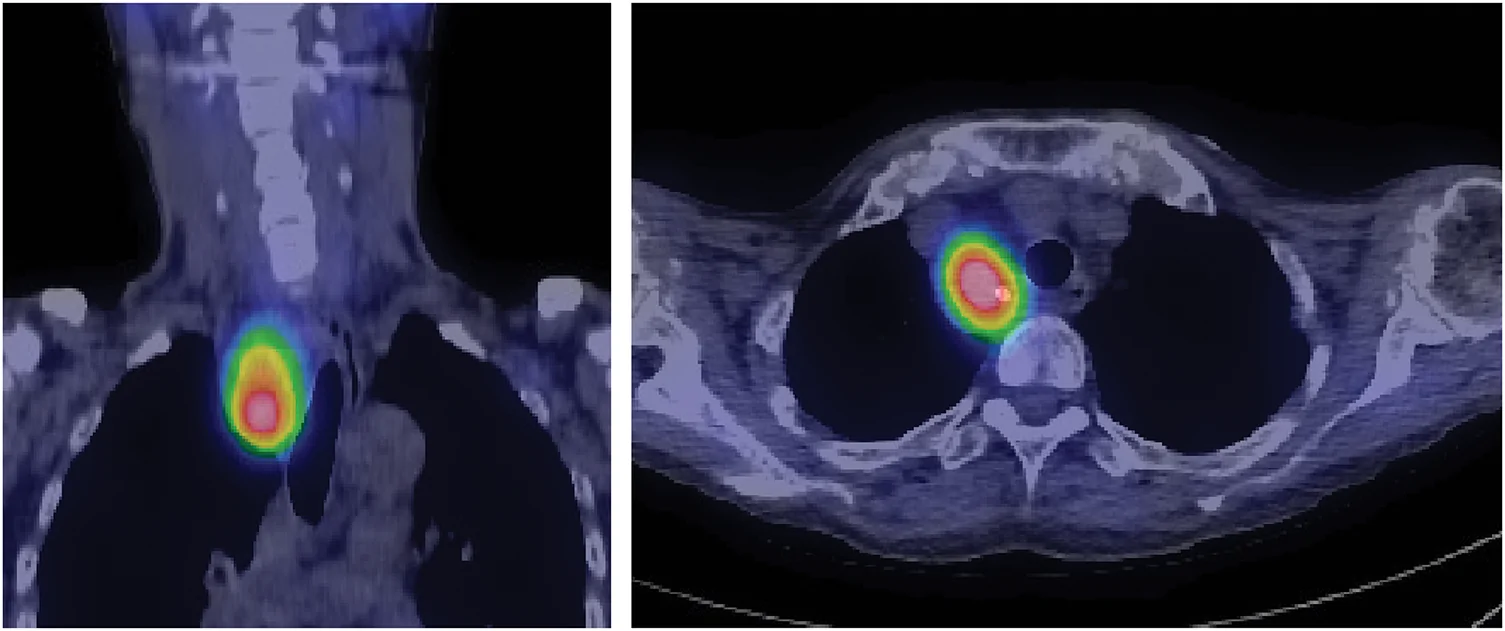
Methoxyisobutylisonitrile scintigraphy demonstrated tracer uptake in a 3-cm calcified mass extending from the region of right thyroid lobe and into the superior mediastinum. Uptake was observed in both the early and late phases, suggesting an ectopic parathyroid adenoma.

Given that the serum Ca level exceeded 12 mg/dL, the iPTH level was >1000 pg/mL, and CT demonstrated irregular skeletal sclerosis throughout the body, parathyroid carcinoma was strongly suspected. As surgical resection is the standard treatment for PHPT and capsular injury in malignant parathyroid tumors carries a high risk of tumor seeding, en bloc resection was planned. However, successful en bloc resection required confirmation that the tumor could be safely dissected within the mediastinum. A thoracoscopic approach was therefore selected because a cervical approach was expected to provide limited visualization and increase the risk of capsular injury, whereas median sternotomy would have been considerably more invasive.

### Operative findings

Surgery was initiated with the patient in the left lateral decubitus position under one-lung ventilation. Intraoperative nerve monitoring was not used. Five thoracoscopic ports were inserted along the anterior and mid-axillary lines at the 3rd, 5th, and 7th intercostal spaces (**Fig. 3A**). The right lung was collapsed to create a pneumothorax and provide an adequate operative field.

**Fig. 3:**
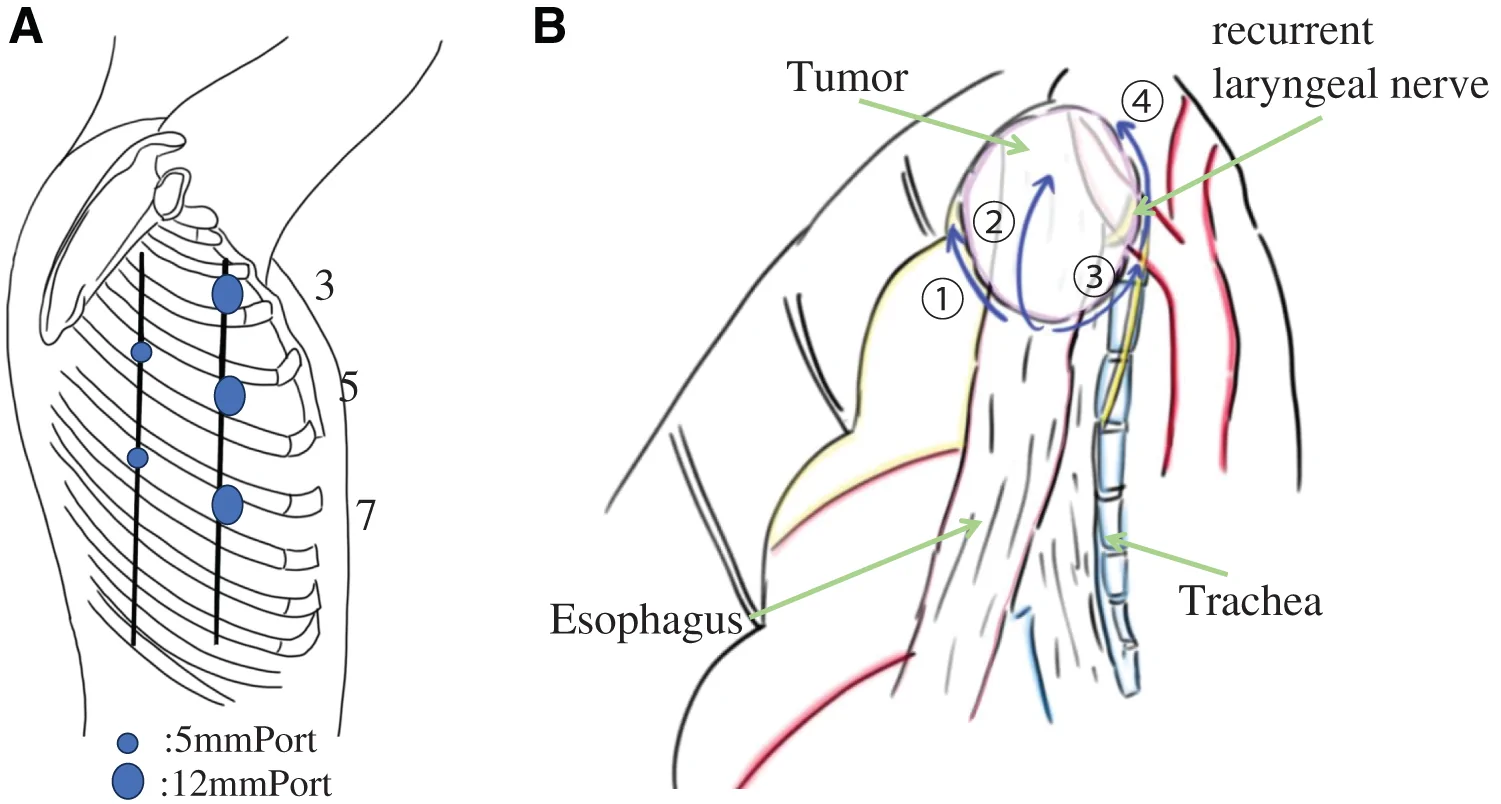
(**A**) Placement of the 5 thoracoscopic ports along the anterior and midaxillary lines at the 3rd, 5th, and 7th intercostal spaces. (**B**) Surgical procedure.

The tumor was carefully retracted and thoracoscopically mobilized from the trachea and esophagus without capsular injury, and dissection was extended to the level of the right subclavian artery. After adequate mobilization, the patient was repositioned supine, and a cervical approach was used to guide the tumor from the mediastinum (**Fig. 3B**). The right recurrent laryngeal nerve was identified and preserved during the dissection (**Fig. 4**). The tumor was removed en bloc together with a portion of the right thyroid lobe while carefully avoiding capsular rupture.

**Fig. 4:**
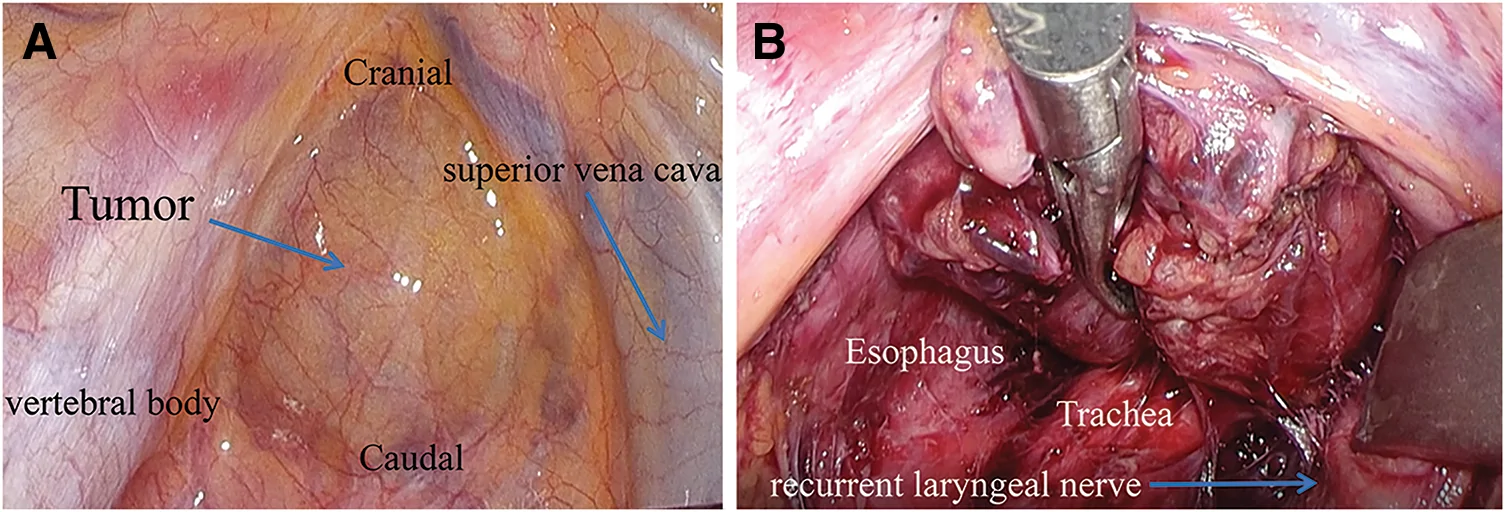
(**A**) A pleura-covered mass was identified in the superior mediastinum between the vertebral body and the superior vena cava. (**B**) The tumor was dissected away from the trachea and esophagus during the thoracoscopic procedure. The recurrent laryngeal nerve was identified and preserved.

The serum Ca level decreased from 10.6 mg/dL on the day of surgery to 9.3 mg/dL on POD 1, whereas iPTH level decreased from 119 to 17.4 pg/mL. On POD 3, the patient developed pneumonia, which resolved with antibiotic therapy. The patient was discharged on POD 9. Pathological examination revealed no mitotic figures (0/20 high-power fields), and neither tumor necrosis nor vascular invasion was observed. The MIB labeling index was low (3%–5%), and immunohistochemical analysis showed that most tumor cells were negative for p53. Additionally, tumor cells lacked expression of the retinoblastoma 1 (RB1) protein (**Fig. 5**). These findings indicated low proliferative activity and a lack of features typically associated with high-grade malignancy. Based on these pathological findings, the final diagnosis was parathyroid adenoma. Accordingly, the patient was scheduled for routine postoperative follow-up at 6 months.

**Fig. 5:**
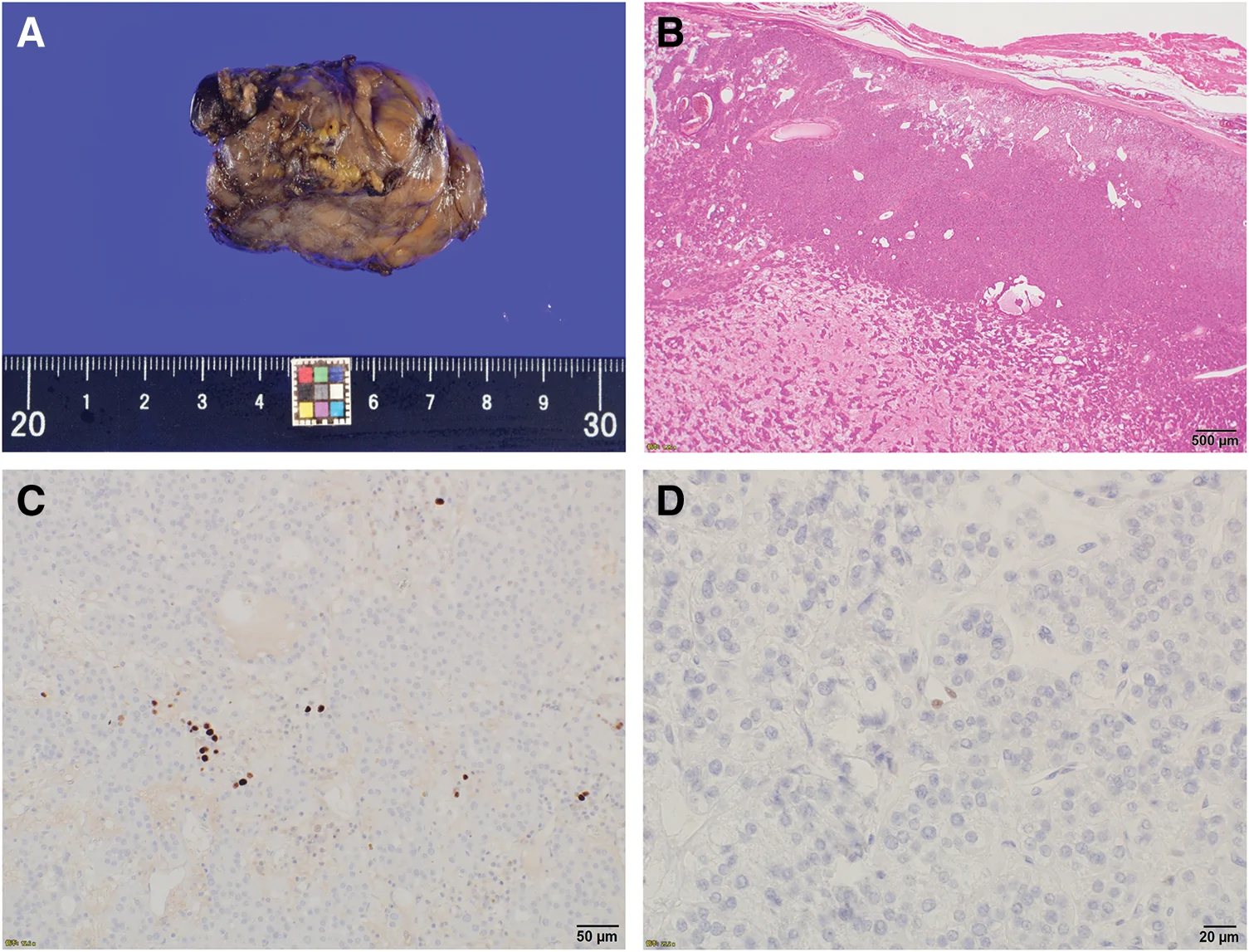
(**A**) Macroscopic findings of the resected specimen. A light brown, solid tumor measuring 54 × 32 × 31 mm^3^ with central cystic changes. (**B**) Hematoxylin and eosin staining showed no prominent mitotic figures. (**C**) The MIB labeling index was low (3%–5%). (**D**) The majority of tumor cells were negative for p53.

## DISCUSSION

Most parathyroid tumors are adenomas and account for the majority of PHPT cases. In contrast, parathyroid carcinoma is rare, representing only 0.3%–5.6% of PHPT cases.^2)^ The standard treatment for PHPT is surgical resection. Although simple tumor excision is generally sufficient for benign parathyroid tumors, en bloc resection with the surrounding tissues is recommended when parathyroid carcinoma is suspected. Intraoperative capsular injury may lead to tumor seeding or an R1 resection (microscopic residual tumor at the resection margin), both of which significantly increase the risk of local recurrence.^8)^

In the present case, the tumor extended deeply into the upper mediastinum, and preoperative imaging raised concerns regarding its proximity to the recurrent laryngeal nerve, subclavian artery, and surrounding mediastinal organs. Therefore, we first performed a thoracoscopic examination of the mediastinum to confirm that the tumor could be dissected from the surrounding critical structures and safely resected before proceeding with the cervical approach.

Previous reports have generally described beginning with a cervical approach and converting to median sternotomy or open thoracotomy if tumor resection proved difficult. In contrast, the thoracoscopic-first approach adopted in this case may be less invasive than median sternotomy or open thoracotomy. Furthermore, the magnified thoracoscopic view provides superior visualization of the tumor margins and adjacent critical structures, including the recurrent laryngeal nerve and subclavian artery, thereby facilitating meticulous dissection and potentially reducing the risk of injury to surrounding organs.

The key clinical features of parathyroid carcinoma include 1) a palpable neck mass, 2) osteitis fibrosa, and 3) marked hypercalcemia, typically with serum Ca levels exceeding 12–14 mg/dL.^1)^ In the present case, preoperative CT revealed that the parathyroid tumor extended from the thyroid into the superior mediastinum, with poorly defined borders adjacent to the trachea and esophagus. When planning en bloc resection, determining whether the tumor can be safely separated from the surrounding tissues without causing capsular injury is essential.^5,9)^ Although several mediastinal parathyroid tumors can be removed using a cervical approach, this is not feasible in all cases.^10,11)^ Previous reports have described the successful thoracoscopic resection of ectopic mediastinal parathyroid tumors. Therefore, when a parathyroid tumor extends into the mediastinum, a thoracoscopic approach may be valuable for evaluating the feasibility of en bloc resection and enabling precise dissection of the tumor under magnified visualization, thereby reducing the risk of capsular rupture.^12,13)^

## CONCLUSIONS

We report a case of a parathyroid tumor extending from the thyroid into the mediastinum that was successfully resected using a thoracoscopic-first approach. This strategy enabled safe mobilization of the tumor from the trachea and esophagus without capsular injury, facilitating complete en bloc resection. A thoracoscopic-first approach should be considered for selected patients in whom complete resection is expected to be difficult through a cervical approach alone.

## DECLARATIONS

### Availability of data and materials

The datasets supporting the conclusions of this article are included within the article.
